# Evaluation of efficacy and pharmacokinetics of novel fluralaner tablet formulation (WellPet™) in dogs

**DOI:** 10.3389/fvets.2026.1760950

**Published:** 2026-04-10

**Authors:** Breno Cayeiro Cruz, Marina Belucci Teixeira, Gessica Ariane de Melo Cruz, Juliana Aparecida do Carmo Emidio Moreira da Silva, Milenni Garcia Michels, Caroline Della Nina Pistoni, Marcus Antonio Martins Buso, Ferdinando Nielsen de Almeida, Igor Renan Honorato Gatto

**Affiliations:** 1 Ourofino Saúde Animal Ltd., São Paulo, Brazil; 2 Graduate Program, School of Veterinary Medicine and Animal Science, São Paulo State University (Universidade Estadual Paulista "Júlio de Mesquita Filho"), Botucatu, São Paulo, Brazil

**Keywords:** Acaricide, Ctenocephalides felis, Ectoparasiticide, fluralaner, insecticide, isoxazoline, *Rhipicephalus sanguineus sensu lato*

## Abstract

The increasing use of isoxazolines represents a major trend in the control of ectoparasites in companion animals. WellPet™ (Ourofino Saúde Animal Ltd., Brazil) is a newly developed, highly palatable oral tablet containing fluralaner. This study aimed to characterize the pharmacokinetic profile of this formulation and to evaluate its efficacy against fleas and ticks for up to 45 days following administration. Two complementary studies were conducted. In the pharmacokinetic study, WellPet™ was administered orally to 12 healthy dogs at a dose range of 10 to 22.5 mg/kg, and plasma fluralaner concentrations were measured at predefined time points up to 49 days post-treatment, yielding a peak plasma concentration (Cmax) of 3,763 μg/L, a time to maximum concentration (Tmax) of 2.0 days, an area under the concentration–time curve (AUCtotal) of 53,342.48 μg·day/L, and an elimination half-life (t½) of 17.88 days. In the efficacy study, 12 dogs were randomly allocated into two groups: six animals treated with WellPet™ and six assigned to a negative control group, and were experimentally infested with fleas (*Ctenocephalides felis felis*) and ticks (*Rhipicephalus sanguineus sensu lato*) at scheduled time points. Post-treatment evaluations demonstrated sustained efficacy above regulatory thresholds, with tick efficacy remaining at 100% from Day 1 to Day 28 and exceeding 97% throughout the 45-day observation period (99.5% on Day 35, 98.1% on Day 42, and 97.98% on Day 45), and flea control reaching 100% efficacy on all post-treatment evaluation days. These findings demonstrate that the proposed label dose of 10 to 22.5 mg/kg achieves plasma concentrations sufficient to support robust and sustained clinical efficacy for at least 45 days, and that the integration of pharmacokinetic and efficacy data provides a mechanistic basis for the observed parasitological outcomes. This optimized 45-day retreatment interval represents a safe and effective alternative to conventional fluralaner regimens, offering reduced dosing frequency, improved owner compliance, and strategic advantages for ectoparasite control and companion animal health management.

## 1 Introduction

The control of ectoparasites in companion animals, mainly dogs and cats, is essential for both animal and public health, given the ability of these parasites to act as vectors of zoonotic and non-zoonotic diseases (1). It is clear that parasite-borne illnesses in humans may be transmitted by their pets, and control measures should involve companion animals to increase their effectiveness. Current trends suggest that multimodal approaches for responsible control of companion animal parasites deserve dedicated attention. The use of parasiticides remains today’s cornerstone for mitigating and controlling the animal and human health threats posed by companion animal parasites (2).

In recent years, the introduction of novel molecules has expanded the range of therapeutic options available, offering enhanced efficacy and safety in the management of flea and tick infestations. In this context, the introduction of the isoxazoline class, mainly represented by fluralaner, afoxolaner, sarolaner and lotilaner (but also tigolaner, esafoxolaner, umifoxolaner, and, for use against agricultural pests, fluxametamide, isocycloseram and fluoxapiprolin, among others), stands out, offering systemically active compounds that function as antagonists of GABA (gamma-aminobutyric acid)-gated and glutamate-gated chloride channels (3). Introduced to the Brazilian veterinary market in the mid-2010s, the first active ingredients approved for use in companion animals were fluralaner, sarolaner, and afoxolaner, which act as systemic insecticides and acaricides (4). These drugs exhibit rapid absorption, reaching peak plasma concentrations within 24 h, with high binding to plasma proteins (>99%) (5), which limits the free fraction and results in only minimal distribution to other tissues such as the liver, kidneys, muscles, and adipose tissue, and are primarily eliminated via feces (6).

Fluralaner stands out within this class due to its unique pharmacokinetic (PK) profile. While other isoxazolines are typically indicated for monthly administration (7, 8, 9), fluralaner can provide efficacy for up to 12 weeks (10). This extended efficacy is directly correlated with the administered dose and is attributed to the long plasma elimination half-life, ranging from 12 to 15 days following oral administration (11, 12), positioning it as one of the leading therapeutic alternatives currently available for the treatment of tick and flea infestations in dogs.

The clinical performance of orally administered drugs is influenced not only by their intrinsic pharmacological properties but also by factors that affect oral absorption and systemic exposure, such as physicochemical characteristics, excipients, and manufacturing processes. Variations in these parameters may lead to meaningful differences in drug absorption profiles, even when the dose of the active ingredient is the same. In this context, characterizing the pharmacokinetic behavior of a formulation is essential to support the interpretation of its clinical efficacy and to ensure consistent therapeutic performance.

The present study aimed to evaluate the efficacy and pharmacokinetics of WellPet™ (Ourofino Saúde Animal Ltd.), a novel highly palatable fluralaner tablet administered at a dose of 10 to 22.5 mg/kg, as a therapeutic alternative for ectoparasite control in preclinical trials in dogs. The product is described as highly palatable based on internal palatability studies conducted by the manufacturer, which demonstrated voluntary consumption of the tablets by dogs. To achieve this objective, the investigation was structured into two complementary and integrated components: a pharmacokinetic study designed to characterize systemic exposure following administration of the reduced-dose formulation, and an ectoparasite efficacy study conducted subsequently to determine whether the level of exposure achieved was sufficient to sustain therapeutic activity throughout the proposed dosing interval.

## 2 Materials and methods

Two independent studies were conducted, both approved by their respective Animal Use Ethics Committees (CEUA) prior to the initiation of experimental procedures. The preclinical plasma pharmacokinetics study was approved under protocol CE-ALI-008/23, and the preclinical tick and flea efficacy study under protocol 001/2023. All procedures strictly followed current Brazilian legislation.

The studies adhered to the principles of the 3Rs, as established by Brazilian regulations, ensuring that “the number of animals used in a project and the duration of each experiment shall be the minimum necessary to produce conclusive results” (13), and that “reducing the number of animals used must not come at the expense of increased suffering of individual animals or compromise the reliability of the results” (14).

Prior to the execution of any experimental procedure, the person responsible for the animals was informed of all planned steps and authorized their participation by signing an Informed Consent Form.

All animals were confirmed to be healthy based on laboratory tests and physical examinations conducted on Day −7 and Day −1 (7 and 1 days before treatment, considered to be Day 0), and were in good sanitary and nutritional condition. None of the animals were undergoing concurrent treatment with other medications, nor had they received any of the active ingredients under investigation within the previous 120 days, or any other drug or vaccine within the last 15 days.

### 2.1 Preclinical pharmacokinetics study

Twelve adult mixed-breed dogs, aged between 1 and 6 years old (mean age of 3.92 years) and weighing from 9.5 to 23.7 kg (mean weight of 14.87 kg), were included in the study, with an equal distribution of males and females.

The administered dose of fluralaner from the WellPet™ formulation ranged from 10 to 22.5 mg/kg, consistent with the labeled dosage recommendations and the fixed-tablet strengths available (45 mg, 100 mg, 200 mg, 400 mg, and 560 mg), which naturally generate variation in mg/kg according to individual body weight. Dogs weighing 4.6–10.0 kg received the 100 mg tablet; those weighing 10.1–20.0 kg received the 200 mg tablet; and animals above 20.1 kg received the 400 mg tablet. All animals received a single oral dose of WellPet™.

Plasma samples for the pharmacokinetic assessment of fluralaner were collected at the following time points: 2 h (h), 4 h, 8 h, 1 day (d), 2 d, 3 d, 4 d, 7 d, 14 d, 21 d, 28 d, 35 d, 42 d, and 49 d. On the day of treatment (D0), each animal received a portion of wet food approximately 15 min before administration, following evidence that the absorption of fluralaner is enhanced when given with food (15). To avoid dietary bias, animals were gradually adapted to this feeding routine for 7 days prior to treatment (D-7 to D-1).

At each sampling point, approximately 4 mL of blood were collected by venipuncture using sterile disposable syringes and needles. The blood was immediately transferred into tubes containing sodium citrate as anticoagulant, centrifuged right after collection, and the resulting plasma was aliquoted and stored frozen. All samples were shipped under controlled conditions to an accredited external analytical laboratory for the determination of fluralaner concentrations.

Quantification of fluralaner in canine plasma was performed by liquid chromatography coupled with tandem mass spectrometry (LC–MS/MS) operated in positive electrospray ionization mode. The contracted laboratory employed a previously validated analytical procedure developed in accordance with international guidelines for bioanalytical method validation, including assessments of selectivity, linearity, precision, accuracy, stability, and absence of carry-over. Calibration curves were prepared in matrix and covered the validated analytical range of 5 to 420 μg/L, with a lower limit of quantification of 5 μg/L. Analyses were conducted using Multiple Reaction Monitoring (MRM) transitions specific for the analyte, and each analytical batch included calibration standards and quality control samples to verify method performance and system suitability. Plasma samples were processed following the laboratory’s validated extraction procedure.

Pharmacokinetic parameters were calculated using the complementary software package PKSolver (16), developed in Visual Basic for Applications (VBA) and implemented within Microsoft Excel®. Non-compartmental analysis was applied to the individual concentration–time data obtained for each dog. The pharmacokinetic results generated from this study provided the biological basis for interpreting the subsequent ectoparasite challenge findings.

### 2.2 Preclinical tick and flea efficacy study

The study was conducted using a total of 12 mixed-breed dogs, aged between 1 and 5 years old (mean age of 2.42 years) and weighing from 8.9 to 21.5 kg (mean weight of 14.70 kg), divided into two experimental groups: six animals treated with WellPet™ and six animals assigned to the Negative Control Group. To ensure proper adaptation, a 21-day acclimatization period was observed prior to the start of procedures.

A randomized block design was adopted, and the study was conducted in a blinded manner by an independent veterinary team from a Contract Research Organization (CRO), not under the direct authority of the sponsor, in order to minimize potential biases. On Day −7, the first infestation was performed, and 48 h later (Day −5), parasite counts and removals were carried out for ranking purposes and to confirm the dogs’ ability to retain fleas and ticks. To be selected, dogs had to retain at least 25% of the parasites used in the infestation (i.e., 25 live fleas and 12 live attached ticks). To preserve study blinding, the researcher responsible for randomization and treatment did not participate in post-treatment data collection. Similarly, personnel responsible for data collection were unaware of the treatment groups and interventions applied.

Similar to the Pharmacokinetics study, the efficacy trial also employed the available tablet presentations within the recommended dose range. However, animal selection prioritized individuals at the upper limit of each weight category, resulting in a mean administered dose of 16.95 mg/kg across the study population. Animals were artificially infested with fleas (*Ctenocephalides felis felis*) and ticks (*Rhipicephalus sanguineus sensu lato*), originating from colonies maintained by the research center where the study was conducted.

Infestations were scheduled to occur in the animals’ housing areas (kennels/pens) on Days −7 and −2 (pre-treatment), and subsequently on Days 5, 12, 19, 26, 33, and 40 post-treatment. Each infestation consisted of approximately 100 adult fleas and 50 adult ticks, aged up to 14 days, unfed, and with a 50:50 male-to-female ratio. Fleas were applied to the dorso-lumbar-sacral region, and ticks near the atlanto-occipital joint. After application, each dog was restrained for at least 1 min and kept calm for approximately 30 min.

Post-treatment evaluations included inspections, counts, and removals conducted on Days 1 (without removal), 2, 7, 14, 21, 28, 35, and 42 after treatment. For parasite counts, dogs were carefully combed over the entire body in the opposite direction of hair growth using a comb with 11 to 13 teeth per centimeter. Removed parasites were recorded using a specific form and automatic counters, then immersed in 70% alcohol. The evaluation was considered complete only after five continuous minutes without the detection of new parasites. Removed ticks were categorized as live unattached, live attached, dead attached, and dead unattached.

Efficacy calculations were based on the number of live fleas and live attached ticks, following the guidelines provided by the World Association for the Advancement of Veterinary Parasitology (WAAVP). The percentage efficacy was determined using the following formula:


$$\text{Efficacy}\;(\%)=\frac{\mathrm{Mc}-\mathrm{Mt}}{\mathrm{Mc}}\;x\;100$$

In this formula, Mc represents the arithmetic mean of the live parasite count in the negative control group, and Mt. represents the arithmetic mean of the live parasite count in the group treated with WellPet™ (17).

Parasite count data were assessed for normality using the Shapiro–Wilk test. If a normal distribution was confirmed (parametric data; *p* > 0.05), flea and tick counts between groups at each time point were compared using analysis of variance (ANOVA). In cases where data were non-parametric (*p* ≤ 0.05), comparisons were performed using the Kruskal–Wallis test. A significance level of 95% (*p* ≤ 0.05) was adopted for all statistical analyses. Data analysis was performed using GraphPad Prism statistical software.

## 3 Results

### 3.1 Preclinical pharmacokinetics study

Dogs treated with WellPet™ received an average dose of 13.23 mg/kg (10.42 to 16.88 mg/kg) of fluralaner per kilogram of body weight, corresponding to approximately 58.78% (46.31 to 75.02%) of the maximum recommended dose.

During the evaluation of individual concentration–time profiles, two animals were identified as outliers: one exhibiting an atypically high Cmax value and another showing a divergent Tmax. These profiles were inconsistent with the overall dataset and were excluded from the pharmacokinetic analysis to ensure data robustness and internal consistency. Consequently, the final pharmacokinetic calculations were based on the remaining 10 animals. All pharmacokinetics values reported here correspond to the mean parameters derived from this final dataset.

**Figure 1:**
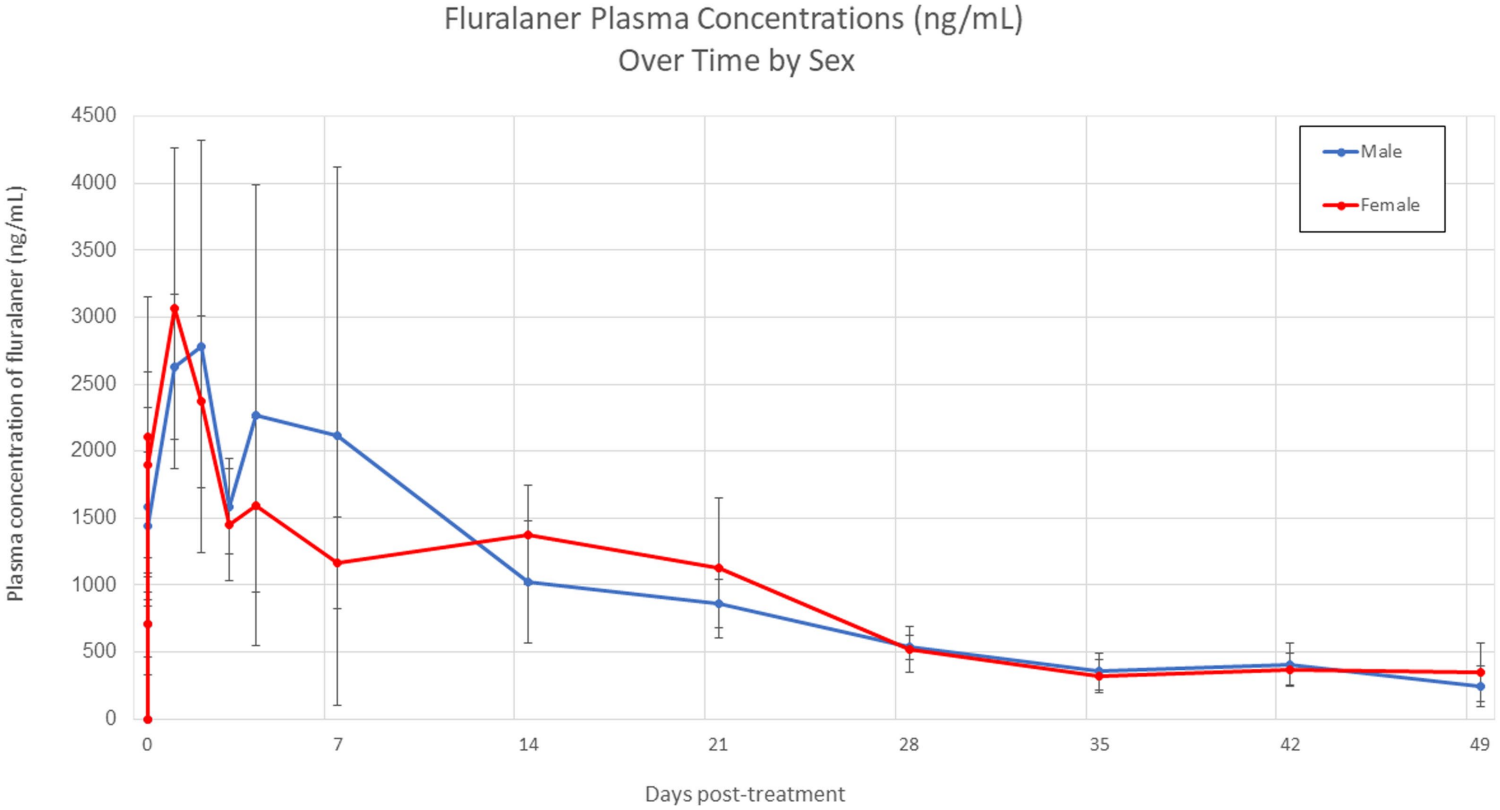
Mean plasma concentration–time profile of fluralaner following oral administration in treated animals by sex.

Based on the concentration–time profiles, the pharmacokinetics curves of male and female dogs were highly similar throughout the evaluation period (Figure 1). Both groups showed comparable peak plasma concentrations within the first hours after dosing, followed by a gradual decline toward Day 49, with overlapping error bars at most time points. This visual similarity was confirmed statistically through an ANOVA using a linear model, which indicated no significant interaction between sex and sampling time (sex × time: *p* = 0.650). Thus, the temporal behavior of fluralaner concentrations was equivalent in males and females, with no evidence of sex-related differences in systemic exposure under the conditions of this study. Consequently, the pharmacokinetics estimates were calculated using data from all animals collectively, without stratification by sex.

Pharmacokinetic parameters were assessed up to 49 days post-administration. Following oral administration of the product, the following values were observed: peak plasma concentration (Cmax) of 3,763 (±1,140) ug/L; time to reach maximum concentration (Tmax) of 2.02 (±2.04) days; area under the curve extrapolated to infinity (AUCinf) of 53,342.48 (±14,913.5) ug/L·day; elimination half-life (t1/2) of 17.88 (±5.75) days; elimination rate constant (Kel) of 0.0415 (±0.0099) day^−1^; apparent volume of distribution (Vz/Fobs) of 0.00071 (±0.0032) (mg/kg)/(μg/L); and apparent clearance (Cl/Fobs) of 0.00028 (±0.00010) (mg/kg)/(ug/L)/day.

**Figure 2:**
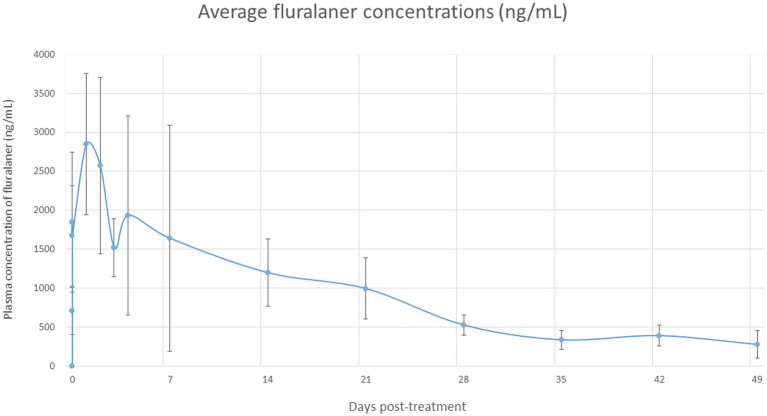
Mean plasma concentration–time profile of fluralaner following oral administration in treated animals.

Pharmacokinetic parameters were derived from individual concentration–time profiles, whereas Figure 2 represents mean plasma concentrations over time. These two approaches provide complementary information regarding systemic exposure and variability among animals.

### 3.2 Preclinical tick and flea efficacy study

Tick and flea counts on Day −5 showed no statistically significant differences between groups, confirming the effectiveness of the randomization process. However, on all subsequent post-treatment days (Day +1 to Day +45), the number of live fleas and live attached ticks in the control group was significantly higher (*p* < 0.05) compared to the treated group, confirming the statistically significant reductions provided by the treatment.

**Figure 3:**
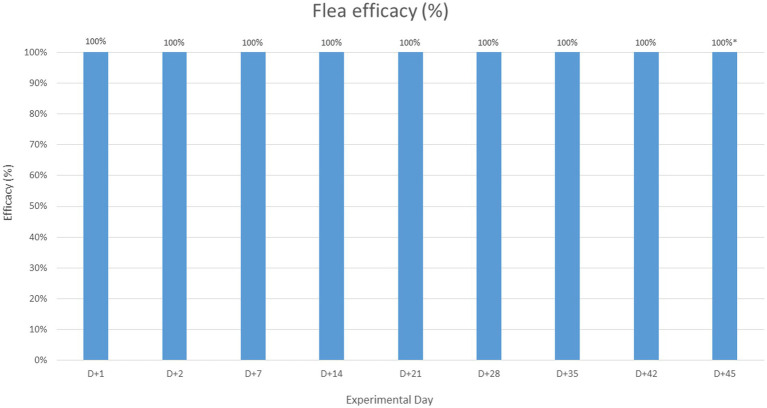
Calculated efficacy on each experimental day. * Statistically estimated data using a type 1 sigmoidal logistic function, based on the values collected on Day +49.

**Figure 4:**
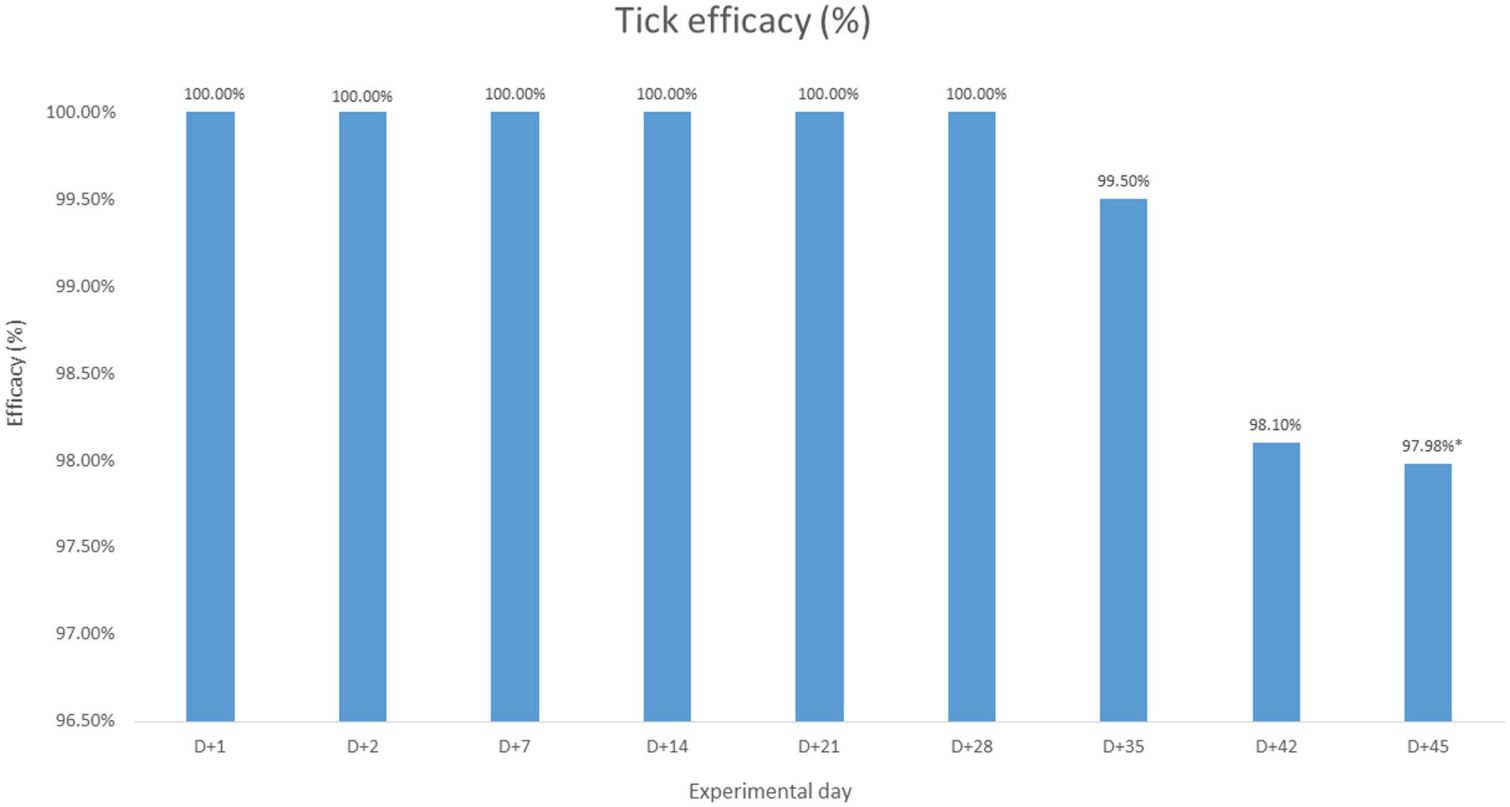
Calculated efficacy on each experimental day. * Statistically estimated data using a type 1 sigmoidal logistic function, based on the values collected on Day +49.

In the treated group, no live fleas were found throughout the entire post-treatment period, indicating 100% efficacy against fleas during this period (Figure 3). Regarding tick counts, no live attached ticks were observed in the treated animals up to Day +28, demonstrating 100% efficacy against ticks during this interval. From Day +35 to Day +45, tick efficacy gradually declined with each weekly assessment, reaching levels below 98% at Day +45 (Figure 4).

## 4 Discussion

Fluralaner is recognized as the isoxazoline with the longest duration of ectoparasiticidal activity, providing quarterly efficacy (12 weeks) at the registered dose range of 25 to 56 mg/kg. This prolonged activity is supported by its pharmacokinetic (PK) profile, characterized by rapid systemic absorption, a Tmax of approximately 1 day after oral administration, and an elimination half-life ranging from 12 to 15 days in dogs (11, 12). These parameters allow plasma concentrations to remain within the therapeutic range for up to 84 days.

In the present study, a novel formulation of fluralaner (WellPet™, Ourofino Saúde Animal Ltd.) was administered at a reduced dose of 10 to 22.5 mg/kg, determined and confirmed through previous studies, and demonstrated a pharmacokinetic profile consistent with systemic absorption and sustained systemic exposure. The observed Cmax of 3,763 ng/mL was comparable to values reported for higher doses (12), while Tmax occurred at approximately 2.0 days, slightly later than the ~1-day Tmax described by Kilp for doses of 12.5, 25, and 50 mg/kg. This shift may indicate a slower absorption rate or modified release characteristics associated with the new formulation. The elimination half-life of 17.88 days closely matched previously published data for the 50 mg/kg dose, indicating that the reduced dose maintains similar pharmacokinetic behavior.

**Figure 5:**
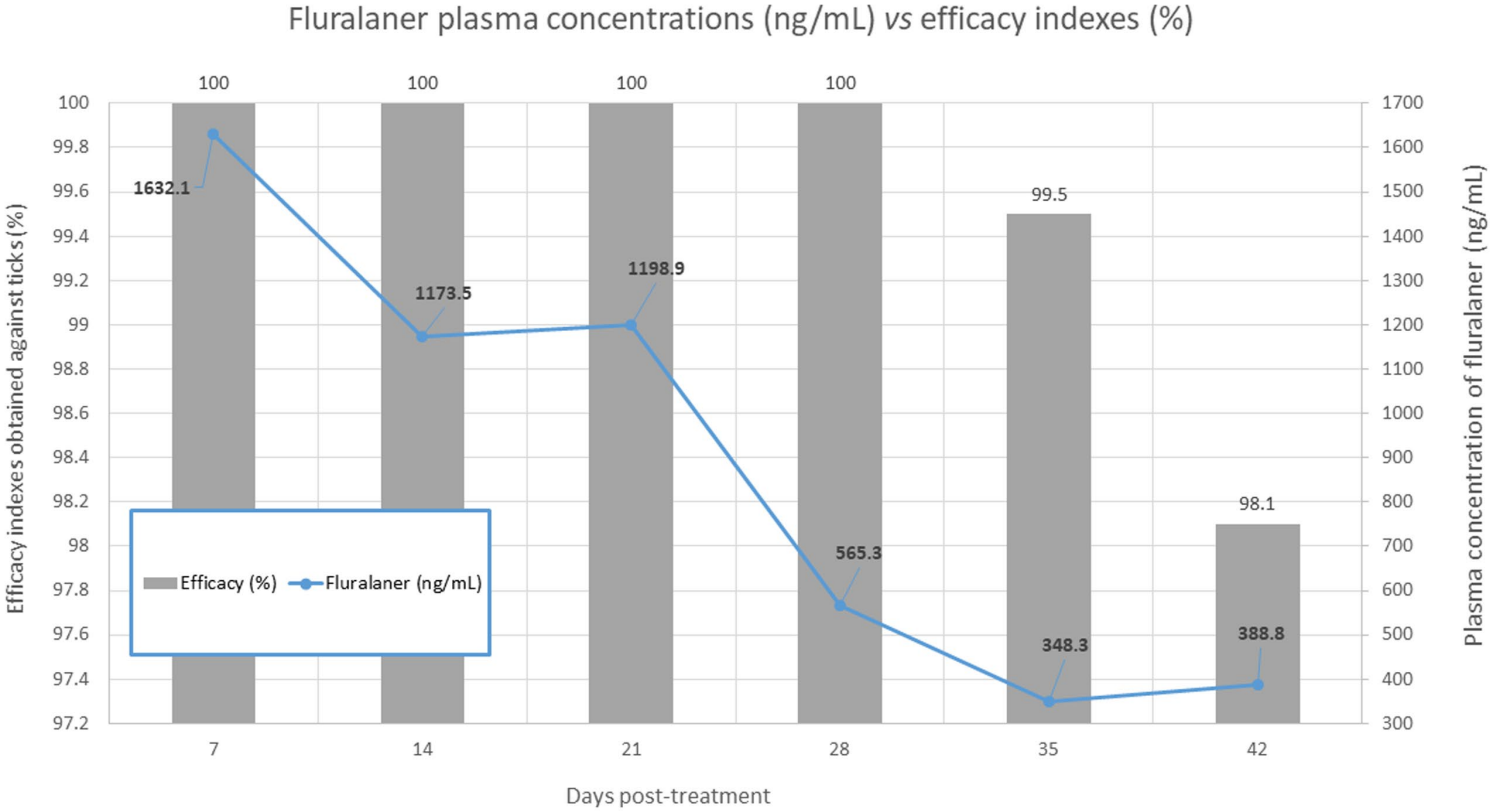
Plasma concentration of fluralaner (ng/mL) and corresponding efficacy (%) against *Rhipicephalus sanguineus* s.l. and *Ctenocephalides felis felis* over time following oral administration of WellPet™ at 10–22.5 mg/kg.

The integration of pharmacokinetic and efficacy data provides a mechanistic explanation for the clinical performance of WellPet™. As shown in Figure 5, plasma fluralaner concentrations remained within the therapeutic range throughout the 45-day evaluation period, coinciding with sustained efficacy against both *Rhipicephalus sanguineus sensu lato* and *Ctenocephalides felis felis*. When compared with previously published pharmacokinetic profiles (12), the concentration–time curve observed in the present study demonstrates behavior similar to that reported for the 25 mg/kg dose, despite the lower administered dosage. The mean peak plasma concentration occurred on Day 1 (≈2,850 ng/mL), close to the value estimated for 25 mg/kg (≈3,000 ng/mL), followed by a decline until Day 28 and stabilization thereafter at levels consistent with the therapeutic threshold described by Kilp.

This sustained systemic exposure was directly reflected in the observed parasitological outcomes, with efficacy reaching 97.98% against ticks and 100% against fleas on Day 45, exceeding the minimum regulatory threshold of 90% (17). These findings demonstrate that the pharmacokinetic profile achieved by the reduced-dose formulation is sufficient to support consistent clinical efficacy throughout the proposed dosing interval.

Compared to other isoxazolines, WellPet™ presents a distinct absorption profile consistent with fluralaner. While afoxolaner and lotilaner reach peak plasma levels within hours (2–6 h and 2–4 h, respectively) (7, 8), fluralaner reaches Tmax over days, reflecting a slower and more sustained absorption process.

The sustained efficacy against fleas and ticks observed in the present study is broadly consistent with previously published data for other isoxazolines. Lotilaner achieved 99.5% efficacy against fleas at Day 28 (9), and sarolaner demonstrated ≥99% efficacy against fleas and ticks for at least 35 days in exploratory studies (3). Tick efficacy indices of 97.98% observed at Day 45 were also comparable to those reported for other isoxazolines, particularly when considering the extended evaluation interval. Afoxolaner, for example, showed 91.9% efficacy against *Haemaphysalis longicornis* at Day 30 following oral administration (18), and 94.2% against *Ixodes scapularis* at the same time point (19). Similarly, injectable fluralaner demonstrated sustained reductions in flea and tick counts exceeding 99% over 1 year (10), and maintained efficacy against *Ixodes ricinus* above 90% for up to 394 days (20). These findings demonstrate efficacy comparable to that reported for other isoxazolines within the evaluated period, representing an effective alternative for ectoparasite control over a 45-day period.

The lower dose used in this study may offer a safety advantage by reducing the total chemical burden on the animal’s body and expanding the safety margin (21). The standard commercial dose of fluralaner, ranging from 25 to 56 mg/kg to sustain efficacy for up to 12 weeks (22), have been associated with gastrointestinal signs (vomiting, reduced appetite, diarrhea, flatulence) and general clinical signs (lethargy, polydipsia) (23). The reduced dosage aligns with pharmacological strategies aimed at maximizing efficacy while minimizing adverse events (21, 24); however, direct comparisons of tolerability between dose regimens require dedicated safety studies.

Oral administration also offers practical advantages, such as eliminating common operational failures associated with topical products, including uneven distribution of the active ingredient and reduced efficacy due to bathing and swimming (22). This route ensures homogeneous drug distribution throughout the animal’s body and improves owner compliance with the therapeutic protocol (22). In regions with continuous ectoparasite exposure and a high risk of vector-borne diseases, such as tropical and subtropical environments, consistent year-round protection remains essential (25). Nevertheless, observational studies indicate that delayed retreatment and incomplete dosing schedules are common in routine practice, potentially allowing parasite populations to re-establish and increasing the risk of reinfestation (26).

Although pharmacokinetic and efficacy data indicate sustained clinical activity up to 45 days post-administration, data collection was intentionally limited to this period. The decision to adopt a 45-day retreatment interval aligns with resistance management strategies for ectoparasiticides. Flea and tick resistance is a well-documented phenomenon, driven by high reproductive rates and frequent exposure to subtherapeutic drug concentrations, which may result from operational failures or the gradual decline of systemic drug levels in the host (27). Therefore, maintaining plasma concentrations above the therapeutic threshold throughout the efficacy interval is essential to prevent the survival and selection of partially resistant individuals. The adoption of a 45-day retreatment interval thus represents a prophylactic measure aimed at minimizing the opportunity for selection of new resistant strains, particularly in environments with high infestation pressure.

Several limitations should be acknowledged. The sample size was limited, and two animals were excluded from the pharmacokinetic analysis as outliers, which may have influenced parameter estimates. In addition, efficacy was evaluated under controlled experimental conditions and may not fully reflect field variability in infestation pressure, host characteristics, or environmental factors. Future studies should investigate longer dosing intervals and real-world clinical settings, as well as further explore the exposure–response relationship and the long-term safety profile associated with repeated administration.

## 5 Conclusion

The pharmacokinetic profile of WellPet™ at a dose of 10 to 22.5 mg/kg, together with the maintenance of efficacy above the regulatory threshold, sustaining 97% efficacy for up to Day 45, confirms that this dosage is sufficient to maintain plasma concentrations at levels adequate to ensure clinical effectiveness. This results in robust clinical efficacy for 45 days in the control of fleas (*Ctenocephalides felis felis*) and ticks (*Rhipicephalus sanguineus sensu lato*) populations, as well as in the prevention of vector-borne diseases.

These findings support the use of WellPet™ as a promising alternative to conventional fluralaner formulations, offering a reduced-dose strategy that preserves efficacy while potentially improving safety and treatment adherence. Further studies are warranted to investigate long-term outcomes and potential applications in broader clinical and epidemiological contexts.

## Data Availability

The original contributions presented in the study are included in the article/Supplementary material, further inquiries can be directed to the corresponding author.
