# Supplementary material for: Evaluation of efficacy and pharmacokinetics of novel fluralaner tablet formulation (WellPet™) in dogs
Source: Front Vet Sci. 2026 Apr 10;13:1760950. doi: 10.3389/fvets.2026.1760950 (PMC13106936; doi:10.3389/fvets.2026.1760950)
Supplement: Supplementary file 1 [file Table_1.docx]

Table 01. Mean tick counts, calculated efficacy indexes, and statistical analysis on each experimental day.

| **Experimental Day** | **Control** | **WellPetTM** | **Efficacy (%)** | **p value** |
| --- | --- | --- | --- | --- |
| D-5 | 36,8 (±6,8)A | 36,8 (±6,4)A | - | >1 |
| D+1 | 37,8 (±5,4)A | 0,0 (±0,0)B | 100 | 0,0007 |
| D+2 | 33,2 (±6,5)A | 0,0 (±0,0)B | 100 | 0,0007 |
| D+7 | 44,8 (±4,2)A | 0,0 (±0,0)B | 100 | 0,0007 |
| D+14 | 42,8 (±6,8)A | 0,0 (±0,0)B | 100 | 0,0007 |
| D+21 | 46,3 (±3,9)A | 0,0 (±0,0)B | 100 | 0,0007 |
| D+28 | 42,2 (±6,5)A | 0,0 (±0,0)B | 100 | 0,0007 |
| D+35 | 35,2 (±6,4)A | 0,2 (±0,4)B | 99,5 | 0,0048 |
| D+42 | 35,2 (±5,9)A | 0,7 (±0,5)A | 98,1 | 0,1695 |

Table 02. Mean flea counts, calculated efficacy indexes, and statistical analysis on each experimental day.

| **Experimental Day** | **Control** | **WellPetTM** | **Efficacy (%)** | **p value** |
| --- | --- | --- | --- | --- |
| D-5 | 75,2 (±13,2) A | 75,0 (±10,0) A | - | >01 |
| D+1 | 74,8 (±10,5) A | 0,0 (±0,0) B | 100 | 0,0007 |
| D+2 | 76,7 (±9,5) A | 0,0 (±0,0) B | 100 | 0,0007 |
| D+7 | 87,7 (±6,9) A | 0,0 (±0,0) B | 100 | 0,0007 |
| D+14 | 85,7 (±6,6) A | 0,0 (±0,0) B | 100 | 0,0007 |
| D+21 | 80,0 (±8,5) A | 0,0 (±0,0) B | 100 | 0,0007 |
| D+28 | 76,8 (±4,8) A | 0,0 (±0,0) B | 100 | 0,0007 |
| D+35 | 73,5 (±9,0) A | 0,0 (±0,0) B | 100 | 0,0007 |
| D+42 | 72,3 (±11,5) A | 0,0 (±0,0) B | 100 | 0,0007 |
